# Increasing access to pelvic health education for women in underserved communities: a mixed-methods study

**DOI:** 10.1093/heapro/daae180

**Published:** 2024-12-06

**Authors:** Alexis Gillett, LaVona Traywick, Kara LaGorio, Anna Dold

**Affiliations:** Alice Walton School of Medicine, 805 McClain Rd., Suite 8000, Bentonville, AR 72712, USA; School of Physical Therapy, Arkansas Colleges of Health Education, 7000 Chad Colley Blvd, Fort Smith, AR 72916,USA; Arkansas College of Osteopathic Medicine, Arkansas Colleges of Health Education, 7000 Chad Colley Blvd, Fort Smith, AR 72916, USA; Arkansas College of Osteopathic Medicine, Arkansas Colleges of Health Education, 7000 Chad Colley Blvd, Fort Smith, AR 72916, USA

**Keywords:** pelvic health, health education, women’s health, underserved communities

## Abstract

It is essential to provide the community with evidenced-based care to optimize healthcare outcomes; more specifically, women in underserved communities with limited access to healthcare services. The purpose of this research was to determine the effectiveness of a single virtual movement-based pelvic health education session on women’s pelvic health knowledge, adherence to performing pelvic exercises and confidence in performing the exercises. Forty-two female participants were recruited in Western Arkansas. Participants completed an online pre-session (pre) questionnaire and then received a single virtual education session led by a healthcare provider trained in pelvic health. A post-session (post-1) questionnaire was completed by each participant as well as a 1-month follow-up (post-2) questionnaire. Thirty-five women completed all three questionnaires and pelvic health knowledge, adherence to pelvic floor exercises and confidence in performing pelvic floor exercises were assessed. After the participants completed the post-2 questionnaire, they were invited for a semi-structured interview and 13 women participated. Results of this study suggest pelvic health knowledge increased after a single session of movement-based education, and this was retained 1 month later. Adherence and confidence to perform the exercises did not change; however, learning about pelvic health in a virtual movement-based method was a positive experience and initiated an eagerness to learn more about pelvic health. Pelvic health education can be implemented in a virtual approach to engage women in underserved communities to learn more about pelvic health through a positive experience for an educational baseline to gain healthcare autonomy.

**Contribution to Health Promotion**

- Pelvic health education is inadequate in rural and underserved communities leading to health inequities for pelvic health care for women.
- A virtual approach to pelvic health education can minimize health inequities through accessibility.
- A single virtual movement-based pelvic health education can increase knowledge on the topic.
- Reaching women in underserved regions through a virtual pelvic health session may improve healthcare autonomy and provide a positive experience.

## BACKGROUND

Providing evidence-based care is essential to optimize healthcare outcomes, and reaching women in underserved communities is crucial to providing such care. Underserved communities are populations without adequate access to medical care, including blue-collar, rural and low-literacy groups (National Institutes of Health, 2024). Pelvic floor anatomy and function is not typically covered in general education in underserved communities which can cause discomfort and avoidance for women broaching this topic with their healthcare provider; in addition, women lack the necessary resources for preventative care for pelvic dysfunction (Mandimika *et al*., 2014; de Andrade *et al*., 2018; Vasconcelos *et al*., 2019). Common pelvic floor dysfunctions include pelvic pain, urinary and bowel incontinence and pelvic organ prolapse (Anil, 2023). Such dysfunctions can decrease individuals’ ability to participate in activities of daily living and significantly hinder their health outcomes. Barriers to healthcare can minimize the opportunity for women to receive adequate pelvic health education (Vasconcelos *et al*., 2019; Jackowich *et al*., 2021). Without proper resources to make educated decisions, they are bound to their current restricted understanding (Vasconcelos *et al*., 2019; Jackowich *et al*., 2021). Furthermore, if women believe their pelvic health symptoms to be ‘normal’ they are less likely to seek help from a healthcare provider (Tinetti *et al*., 2018). Disrupting inequity of women in underserved communities who are not provided an adequate pelvic health education is a necessity in the forward movement of women in society.

An approach to treating and preventing pelvic floor dysfunctions is using movements to target the musculature of the pelvis (de Freitas *et al*., 2018; Tinetti *et al*., 2018). Effective treatment for pelvic floor dysfunctions is dependent on patients’ consistent adherence to the regime (de Freitas *et al*., 2018; Navarro-Brazález *et al*., 2021). Online modalities are useful to educate patients remotely and to approach sensitive topics like pelvic health (Jackowich *et al*., 2021; Morrison *et al*., 2022; Sjöström *et al*., 2013). Increasing patient education on pelvic health may increase women’s comfort in addressing concerns with their healthcare providers and provide women in underserved communities with resources for care (Vasconcelos *et al*., 2019; Jackowich *et al*., 2021).

While pelvic health education is important to address, literature is lacking in evidence for gaining pelvic health knowledge, confidence related to this knowledge and the inclusion of utilizing movement-based intervention to bridge this gap (Jackowich *et al*., 2021; Morrison *et al*., 2022; Sjöström *et al*., 2013; Vasconcelos *et al*., 2019). More research is needed to understand how to reach women in underserved communities on the topic of pelvic health to equip them with the necessary tools. The purpose of this study was to determine the effectiveness of a single movement-based pelvic health education session on general pelvic health knowledge, underactive pelvic floor and overactive pelvic floor knowledge and adherence and confidence to performing pelvic exercises in women through a virtual format. The study also aimed to assess motivators/barriers to adherence and implementation of pelvic exercises. Women lacking access to medical services were identified to address this issue in a region in Western Arkansas.

## METHODS

### Design

This mixed-methods study was conducted by researchers at a higher education institution in Western Arkansas, USA and was approved by the affiliated Institutional Review Board (IRB #2022-0107). A mixed-methods study was chosen to collect objective data as well as gain a greater understanding of women’s thoughts and perspectives on pelvic health education and barriers faced. The Transparent Reporting and Evaluations with Nonrandomized Designs guidelines were used for appropriate reporting with the associated statement checklist. The TREND checklist can be accessed through the Centers for Disease Control and Prevention website (Centers for Disease Control and Prevention, 2018).

### Participants

Female participants were recruited through purposeful sampling using social media announcements, newsletters and email over a 3-month period. Forty-two participants met the inclusion criteria of female-identifying, 18 years of age or older, ability to speak English or provide their own translator, ability to complete online questionnaires, having access to email and device to participate in a virtual meeting and being local to the region to target an underserved pelvic health population. Participants were excluded if they were pregnant or experiencing any pelvic pain/pelvic issues and did not have permission from their physician to participate in the exercise. Women who met the inclusion criteria were supplied with a package including information about the study, a notebook and pen and images of the movements included in the virtual session to follow along with the presenters.

### Intervention

Participants completed an informed consent form and online pre-session (pre) questionnaire. Once the pre-test was complete, the participants received a link for a single virtual movement-based educational intervention session led by a pelvic health physical therapist. The participants could choose to attend one of five different virtual sessions offered at various times over the course of a year. The virtual session was ~1 h including a combination of pre-recorded and live instruction of movement-based education through various positions, exercises and stretches. The instructor gave explanations for each pose as it relates to pelvic health while infusing information about general pelvic health during the demonstration of the movements. Immediately after the virtual session, the participants completed a post-session (post-1) questionnaire; in addition, 1 month after the session the participants completed a follow-up (post-2) questionnaire. The questionnaire was derived from the Prolapse and Incontinence Knowledge Questionnaire (PIKQ), a previously validated questionnaire, with additional items added for a broader assessment of women’s pelvic health knowledge (Mandimika *et al*., 2014). The questionnaires included a total of 30 questions with 10 questions in the following categories: general pelvic health knowledge, underactive pelvic floor and overactive pelvic floor. All participants who completed the post-2 questionnaire were invited to a semi-structured interview and were offered a yoga mat as an incentive. The participants who volunteered to participate were interviewed by graduate student researchers in a private setting either in-person or virtually and answered open-ended questions regarding overall experience, learning acquisition and implementation of session information. The interviews were audio recorded and ranged from 10 to 30 min. The interviewers did not attend the virtual sessions to minimize bias and promote an environment for the participants to share their thoughts candidly.

### Data synthesis and analysis

Data collected for quantitative and qualitative methods addressed differing questions. The quantitative data were used to measure knowledge gained, adherence and confidence in pelvic floor exercises. Pelvic health knowledge was compared between the three timepoints (pre, post-1 and post-2) using repeated measures ANOVA in SPSS, and a *p*-value of less than 0.5 was used to determine statistical significance. Repeated ANOVA was an appropriate statistical test to utilize due to the small, but equal sample sizes (Sullivan *et al*., 2016). Using the Shapiro–Wilk test, data were found not normally distributed; however, the repeated ANOVA was considered an appropriate test to compare the same subjects over time. Adherence to pelvic floor exercises was included in the pre and post-2 questionnaires. Confidence in performing pelvic floor exercises was included in the post-1 and post-2 questionnaires. Adherence and confidence in pelvic floor exercises were compared using a Wilcoxon-signed rank test. Participants who did not complete all three questionnaires were excluded from the data analysis.

The qualitative data were used to assess motivators/barriers to adherence and implementation of pelvic exercise. All interviews were conducted by two of the researchers for consistency. The audio was transcribed by the interviewing researcher. Using the methods outlined by Creswell and Poth (2016) the transcripts were separately coded, with each coder independently assigning codes to identify key concepts and patterns. Once the initial coding was completed, the coders compared their codes to ensure consistency and reliability, discussing any discrepancies to reach a consensus. This process was followed by axial coding, where the relationships between the codes were examined and categories were refined and linked together. Overarching themes were identified by synthesizing the categories. For example, codes for time, schedules, conflicts and routines were linked to the participant’s capacity for completing pelvis floor exercise. Once a pattern was identified for lack of adherence and that category was established, a reoccurring idea of the need for prioritization was observed as a theme.

### Role of the funding source

The funders played no role in the design, conduct or reporting of this study. This study was funded by the researchers’ institution and additional information is described in the Funding section below.

## RESULTS

### Participant characteristics

Forty-two women started the study and 35 completed all of the required components. The participants identified their age range and were represented in each category from 18 to 29 through 70 and older. More than half of the women were between the ages of 18 and 39 (54%) and the majority of the women were Caucasian (85.7%) (Table 1). Most participants had completed a bachelor’s degree or higher (85.7%), had given birth (65.7%) and could always afford things they needed (82.9%) (Table 1). Of the 35 participants who completed the required components, 13 participated in the post-session interview. Additional characteristics of the interviewed participants were not collected for the anonymity of the respondents and to minimize bias when analyzing the results.

**Table 1:** Participant demographic characteristics

Age range	Frequency
18–29	8
30–39	11
40–49	7
50–59	6
60–69	2
70 and over	1
**Highest education**	
Graduate degree	15
Bachelor degree	14
Associate degree	2
Some college, but no degree	4
High school	0
**Race/Ethnicity**	
White/Caucasian	30
Hispanic	1
Asian	1
American Indian or Alaskan Native	1
Multiple races	2
**Able to afford things needed**	
Always	29
Often	6
**Given birth**	
Yes	23
No	12

### Quantitative results

The average overall knowledge scores for the pre, post-1 and post-2 surveys were 64.7%, 92.7% and 92.9%, respectively with 100% representing a perfect score. There was a statistically significant difference between pre/post-1 knowledge and pre/post-2 knowledge (*p* < 0.001); however, there was no difference in knowledge between post-1 and post-2 knowledge (Table 1). When comparing general, underactive and overactive pelvic floor knowledge at pre, post-1 and post-2, the results aligned with the overall knowledge score outcomes regarding statistically significant differences found between pre/post-1and pre/post-2, but no difference between post-1 and post-2 (Table 2).

**Table 2:** Survey comparisons of overall, general, underactive pelvic floor, overactive pelvic floor knowledge for pre, post-1 and post-2 questionnaires

Overall knowledge	Mean difference (absolute)	*p*-value	95% CI
Pre vs post-1	28.0	<0.001	19.8, 36.2
Pre vs post-2	28.3	<0.001	20.1, 36.5
Post-1 vs post-2	0.3	0.996	0, 7.9
**General knowledge**			
Pre vs post-1	31.4	<0.001	22.2, 40.6
Pre vs post-2	31.7	<0.001	22.5, 40.9
Post-1 vs post-2	0.3	1.00	0, 8.9
**Underactive pelvic floor knowledge**			
Pre vs post-1	23.7	<0.001	15.3, 32.1
Pre vs post-2	26.3	<0.001	17.8, 34.7
Post-1 vs post-2	2.6	0.75	0, 5.8
**Overactive pelvic floor knowledge**			
Pre vs post-1	28.9	<0.001	15.8, 42.0
Pre vs post-2	26.9	<0.001	13.8, 40.0
Post-1 vs post-2	2.0	0.93	0, 15.1

*Note:* Pelvic health knowledge was compared between the three timepoints (pre, post-1 and post-2) using a repeated measures ANOVA in SPSS, and a *p*-value of less than 0.5 was used to determine statistical significance.

No statistical difference was found from pre-session to post-2 regarding how often participants performed pelvic floor muscle exercises (mean score of 1.7 and 1.8, respectively) (Table 3). The frequency mean was calculated by converting Likert scale answers into a numerical representation and (0 representing never and 4 representing at least once a day) and averaging the values of participants. In addition, no statistical difference in the overall confidence mean score of participants from post-1 (13.1) to post-2 (12.2) when averaging participants’ responses after converting Likert scale answers into numerical representation (0 representing not at all confident and 4 representing completely confident) (Table 3).

**Table 3:** Adherence and confidence to perform pelvic floor exercises

Adherence to pelvic floor exercises	Mean difference (absolute)	Standard deviation	*P*-value (pre vs post-2)
Pre	1.7	1.69	0.79
Post-2	1.8	1.39	
Confidence to perform pelvic floor exercises	Mean difference (absolute)	Standard deviation	*P*-value (post-1 vs post-2)
Post-1	13.1	3.65	0.69
Post-2	12.3	4.25	

*Note:* For adherence to pelvic floor exercises, frequency mean was calculated by converting Likert scale answers into a numerical representation and (0 representing never and 4 representing at least once a day) and confidence to perform pelvic floor exercises was calculated by converting Likert scale answers into numerical representation (0 representing not at all confident and 4 representing completely confident) and averaging the values for each, respectively. Wilcoxon-signed rank test was used to determine the *p*-value.

### Qualitative results

Thematic analysis of the 13 transcribed interviews indicated that participants found learning about pelvic health a positive experience regardless of prior apprehensions. Participants described having a comprehensive pelvic health baseline knowledge after the intervention that would affect discussions with their primary care provider, but still expressed wanting more information and specific direction for implementation. Many participants also described specific barriers that would prevent them from discussing their pelvic health with their primary care physician despite feeling competent in the subject. Participants repeated the sentiment of a need for prioritization of time and more tailored exercises in order to maintain consistent exercise adherence.

#### Knowledge—comprehensive education

The interviewed participants emphasized their increase in pelvic health knowledge and the benefits of the comprehensive educational approach on this topic. One participant noted, ‘I thought it was all about the tightening and the exercises’ and noted that at the beginning of the session, she felt ‘confused when stretching was mentioned’; however, she understood the reasoning for including the stretches by the end of the session. Another participant emphasized how important she thought it was to learn this information earlier rather than later saying ‘I just wished that they would teach women these things earlier’. Interviewed participants consistently mentioned the educational portion of the intervention provided an easily digestible, yet substantial baseline knowledge of pelvic health. Multiple participants had positive comments regarding the various formats used to present the information. For example, one participant mentioned the benefit of having a pamphlet with pictures to ‘jog your memory’. Recommendations for improvement included allowing more time for note-taking and having access to video presentations after the session as a reference.

#### Adherence—need for prioritization

Another theme of the interviewed participants was a need for prioritization before consistent exercise adherence could be maintained. Participants repeatedly named time constraints as the primary barrier preventing them from consistent exercise implementation. One participant summed up this barrier stating ‘[The] learning bit is great because that’s half the empowerment, but then how do we build this as habits in our lives?’ Nearly every participant stressed understanding the need to work the exercises into their daily routine, but said time restraints prevented them from consistently doing pelvic health exercises. Participants noted the motivation to do these exercises was necessary as adding one more task to an already busy schedule can be daunting when already feeling overwhelmed. A participant shared ‘All [the exercises], they’re really minimal. They don’t take a lot of your time’. The same participant further explained that despite the exercises being ‘minimal’, building the exercises into a routine was her biggest barrier to consistently performing them. Some participants added that acquiring an accountability partner or a habit tracker application to assist them in making this part of their daily routine may be beneficial. One participant summed up their desire to adhere to the exercises noting, ‘If I can make this part of my habits, then I know I’m gonna [feel better]’. Some participants also stated wanting to better prioritize which exercises were most beneficial for their specific pelvic health concerns. One woman, who was pregnant, described wanting a pinpointed set: ‘these five are your top five for pregnancy, or these five are your top five for postpartum… I can’t do all 20 all the time, so what are the best ones to focus on?’ Despite the theme of a need for prioritization, several of the interviewed participants stated that after the intervention they initiated performing pelvic health exercises again after previously stopping or had achieved less pain since learning the movements.

#### Confidence—developing competence

After the intervention, participants felt competent discussing pelvic health, but still had barriers preventing them from discussing it with their primary care provider. Participants expressed the intervention had provided a baseline knowledge on pelvic health topics, with one saying, ‘I would be able to have a more intelligent conversation [right now], versus not having an idea of pelvic floor health’. One participant when asked about her confidence discussing her pelvic health with her primary care physician, remarked ‘after the course, the barriers [to discussing with my doctor] would no longer be on lack of education, but would be more so the relationship that I have with that primary care doctor’. Other participants remarked about their doctor’s gender or past relationship with their provider influencing their willingness to openly discuss their pelvic health.

Increasing participants’ knowledge of pelvic health was an aim of this study; however, questions were raised by participants about their doctors’ knowledge of pelvic health as well. A participant felt that when voicing her postpartum pelvic health concerns to her doctor, he seemed incapable of directing her on the topic. When asked about the material, multiple participants made comments to the effect of recommending the course to other women to develop competence and confidence on the topic. To summarize this concept, a participant stated, ‘Having the education yourself and that knowledge for yourself gives you not only the confidence to question [doctor’s recommendations] but also just the knowledge itself to either avoid going or assist in that conversation with your own physician’.

## DISCUSSION

This mixed-methods study suggested females have a greater knowledge of general pelvic health and pelvic-related dysfunctions after a single session of movement-based education, and knowledge can be retained 1 month later. Virtual movement-based patient education may be effective; however, participants’ knowledge acquisition is not enough for change in behavior. Participants reported developing confidence in discussing their pelvic health but did not display a change in adherence or confidence in performing the pelvic exercises.

Overall, knowledge was gained and retained by the participants through an innovative approach used in this study to deliver information in a community lacking pelvic health resources and accessibility. While knowledge was gained, confidence and implementation of the pelvic exercises did not change. The intervention provided a broad scope of education regarding pelvic health; however, activities tailored to the individual or specific population may provide greater benefits (Dao and Dunivan, 2022). A treatment plan must account for the individual needs of patients to optimize knowledge and promote adherence to build confidence in performing the movements. Women in underserved communities may require both a broad scope of education for baseline pelvic health knowledge as well as condition-specific education for individuals’ needs to be met.

Results of this study may indicate an increase in a participant’s knowledge of pelvic health could be related to increased comfort in discussing the topic with their healthcare provider. Several participants indicated their discomfort in asking questions to their healthcare provider before the study and the role gender difference plays in their pelvic care. The qualitative results provided insight into how the participants felt about their confidence in discussing their pelvic health with other women and healthcare providers after the intervention. Through virtual intervention, women were able to gain knowledge which subsequently may have led to an increased comfort level in discussing their pelvic health with their healthcare provider. An increase in baseline knowledge, discussion of what may be considered a taboo topic and raising awareness about pelvic health could help bridge the gap between patient and provider (Snyder *et al*., 2022).

There were several limitations in this study, including a small sample size. The researchers acknowledge they did not calculate the sample size for power to determine a detectable difference in the results, instead, the researchers accepted whatever sample size was made available by individuals willing to participate. Given the exploratory nature of the study, hypothesis testing was not expected to be the primary result of the study, and therefore, sample size was not paramount. The small sample size in this study may be due to women having discomfort in learning about pelvic health and such discomfort may increase hesitancy for participation. Despite women having a college education and financial security, their baseline scores for the study (64%) indicated a lack of pelvic health knowledge. Mandimika *et al*. (2014) concluded that limited knowledge is widespread among women of all racial/ethnic groups; however, racial disparities in African American women and other women of color have significantly limited knowledge. Furthermore, a recent study found that African Americans do not utilize physical therapy services as quickly as Caucasians (Richter *et al*., 2022). The lack of racial/ethnic diversity in participants for this study may be attributed to the significantly limited knowledge and underutilization of healthcare services including interventions for pelvic health education.

A limitation of the study was a lack of diversity within the participants recruited, thus lacking generalizability to larger populations. The participants were primarily Caucasian, college-educated, and could afford things they needed. Another limitation of the study was the small sample size in an underserved community. The community where the study took place was underserved in regards to inadequate access to medical care, primarily a blue-collar population, and low-literacy groups. The participants, though college-educated with financial resources, still lacked access to adequate medical care, thus being classified as an underserved population.

The researchers recruited participants across multiple platforms, for several months and provided multiple days and times for participation options to accommodate scheduling conflicts. A further limitation of this study was a lack of long-term follow-up with participants. The findings demonstrated the participant’s knowledge, adherence to exercises and confidence after 1 month, but a long-term follow-up of 3, 6 or 12 months may have provided differing results with insight into behavioral factors (Venegas *et al*., 2018). Additional considerations to this study include the researchers utilizing a questionnaire derived from a previously validated questionnaire; however, the questionnaire used for this study has not yet been validated.

## CONCLUSION

In summary, the results of the study showed alternative means of patient education may be effective, but knowledge gained does not equate to a behavior change. Thus, a clinical implication is that virtual pelvic health educational sessions are a viable way to educate underserved communities and have knowledge gain; however, more than one session is needed for behavior change. Future studies should include education for specific pelvic health conditions in underserved communities to improve women’s knowledge, exercise adherence and confidence in optimizing their pelvic health.

## Data Availability

The data underlying this article cannot be shared publicly for the privacy of individuals who participated in the study. The data will be shared on reasonable request to the corresponding author.
